# Integrated Experimental and Theoretical Studies of Stem Cells

**DOI:** 10.1007/s40778-017-0096-2

**Published:** 2017-07-10

**Authors:** Hanna L. Sladitschek, Pierre A. Neveu

**Affiliations:** Cell Biology and Biophysics Unit, European Molecular Biology Laboratory, Meyerhofstr. 1, 69117 Heidelberg, Germany

**Keywords:** Stem cells, Embryonic stem cells, Modeling, Single-cell heterogeneity, Differentiation, Self-organization, Morphogenesis

## Abstract

**Purpose of Review:**

Stem cells have to balance self-renewal and differentiation. The dynamic nature of these fate decisions has made stem cell study by traditional methods particularly challenging. Here we highlight recent advances in the field that draw on combining quantitative experiments and modeling to illuminate the biology of stem cells both in vitro and in vivo.

**Recent Findings:**

Recent studies have shown that seemingly complex processes such as the fate decision-making of stem cells or the self-organization of developing tissues obey remarkably simple mathematical models. Negative feedback loops appear to stabilize cellular states hereby ensuring robust fate decision-making and reproducible outcomes. Stochastic fate decisions can account for the great variability observed in biological systems.

**Summary:**

The study of stem cells is hampered by the necessity to track the fate of a cell’s progeny over time. Confronting experiments with simple predictive models has allowed to circumvent this problem and gain insights from stem cell heterogeneity in vitro to organ morphogenesis.

## Introduction

Stem cells possess the dual ability to either self-renew or differentiate. This fate decision is dynamically controlled to create and maintain a functional tissue derived from a small number of stem cells. Identifying and studying critical factors that influence stem cell behavior requires monitoring the stem cell and its progeny over extended periods of time. Such experiments depend both on the availability of molecular markers to determine the fate of a given cell and tools to fully reconstruct its pedigree. However, accurate lineage tracing is particularly challenging in vivo, where both long-term live imaging and the genetics necessary to identify the relevant cell populations are demanding. In vitro stem cell cultures offer the advantage of their accessibility in order to understand the principle of fate transitions but can suffer from a large degree of heterogeneity.

In the following, we will illustrate a few cases in which a combination of quantitative experiments and modeling has allowed to overcome some of these limitations and to decipher stem cell behavior.

## Embryonic Stem Cell Heterogeneity

Mouse embryonic stem cells (ESCs) are derived from the inner cell mass of the pre-implantation blastocyst. They can self-renew indefinitely in vitro while maintaining intact their capacity to differentiate into derivatives of all three germ layers and germ cells both in vitro and in vivo [1, 2]. The conventional monoculture regime relies on the cytokine leukemia inhibitory factor (LIF) supplemented with serum [3, 4]. Binding of LIF to a complex of LIF receptor (LIFR) and its co-receptor gp130 activates signal transducer and activator of transcription 3 (STAT3) [5] as well as PI3 kinase/AKT [6]. These two pathways are known to promote mouse ESC self-renewal and cell-survival. Paradoxically, LIF-dependent gp130 signaling also stimulates the mitogen-activated protein kinase/extracellular signal-regulated kinase (MAPK/ERK) pathway [7], a driving force of differentiation [8]. The notion of LIF-dependent pluripotency as a metastable state is further supported by the observation that clonal mouse ESCs cultured in a uniform signaling environment exhibit a high degree of cell-to-cell variability in gene expression [9], epigenetic marks [10], colony morphology [11], responsiveness to differentiation cues [12], and chimera contribution potential [13]. It has been argued that mouse ESC heterogeneity in LIF + serum might be a mere culture artifact stemming from an ambiguous signaling environment where cytokines contained in serum bias cells towards commitment whereas LIF instructs self-renewal [14, 15].

We recently addressed the question whether LIF-dependent pluripotency is intrinsically heterogeneous [16••]. Taking advantage of a single-cell fluorescent microRNA reporter, we uncovered that the miRNA miR-142 is bimodally expressed in LIF-dependent pluripotency [16••]. miR-142 expression segregates functionally a self-renewing mouse ESC population which otherwise expresses pluripotency factors at similar levels into a differentiation-competent subpopulation (with low miR-142 levels) or a differentiation-incompetent pool (with high miR-142 levels). Cell sorting experiments showed that both miR-142 states can reconstitute the other one. Fitting a simple reaction kinetics model on the temporal dynamics of culture reconstitution allowed us to precisely determine interconversion rates at the population level, with switching occurring on average once every 12 cell divisions and no difference in proliferation rates between the two states. The same model can be used to determine if state switching is a stochastic process. Indeed, the state composition of single-cell derived cultures grown for durations of the order of the inverse of the measured switching rate will depend on the particular timing of individual switching events. Contrasting simulations with the quantification of state composition in a large number of cultures originating from a single cell sorted in a defined high or low miR-142 state demonstrated that state switching is stochastic and allowed to predict that high miR-142 cells are less clonogenic than low miR-142 cells. Further experiments showed that miR-142 is indeed instrumental in restricting the clonogenicity of mouse ESCs. The bimodal expression of miR-142 can be mechanistically explained by a double negative feedback loop between miR-142 and Kras/Erk activation: miR-142 represses Kras translation while Erk activity represses miR-142 transcription. Only three parameters are sufficient to characterize this system. Non-linearity in the feedbacks creates a bistable system for a wide range of miR-142 production and ERK turnover rates that are compatible with biological constraints. This bistable model was challenged and validated by creating mouse ESCs with broken feedback loops or with a single miR-142 allele. Thus, this bistable system renders LIF-dependent pluripotency intrinsically heterogeneous.

## Studying Stem Cell Transitions

The heterogeneity of self-renewing stem cell populations creates a major challenge in the study of stem cell behaviors. In the example above, the study of miR-142 heterogeneity in mouse ESCs required extensive development of tools to characterize the system. However, heterogeneity is even more pervasive as fluorescent knock-in reporter constructs and single-cell RNA sequencing experiments have demonstrated that a large number of genes are heterogeneously expressed in self-renewing mouse ESCs [9, 17–20], defining a potentially large number of mouse ESC states. The hierarchy and interrelationships between these states are not clear and daunting to address experimentally with current techniques.

Hormoz et al. [21••] offer an elegant approach to this problem. They devise a model named kin correlation analysis (KCA) that relies on the lineage tree and state attribution of individual cells to infer the dynamics of state transitions in a population. Indeed, cells sharing common ancestry are more likely to be found in the same cellular state than unrelated cells. They originally applied a model for reversible state transitions to bacteria populations [22] and now generalize it to irreversible and non-stationary dynamics. The analysis requires the pedigree of the population that can be obtained by simple time lapse imaging in the absence of any markers, circumventing the need of engineered transgenic cell lines. In addition, states of individual cells at the end point of the lineage tree must be determined. Then, the probability to observe cells in the different states with a common ancestor a number of generations ago is computed, building a two-cell correlation matrix. The transition matrix can be inferred from the two-cell correlation matrix while three-cell correlation matrices are used to infer irreversible dynamics. KCA can be used as well in the case of probabilistic state assignment and obviously applies only to states that persist longer than a cell division. They validate their method using a fluorescent reporter for Esrrb, a gene known to be heterogeneously expressed in mouse ESCs. Then using single molecule RNA fluorescence in situ hybridization (smFISH), they infer the transition network between five states defined by the levels of three genes Esrrb, Tbx3, and Zscan4. Interestingly, a network with chain-like dynamics can account for the experimental data. This limited number of transitions signifies that mouse ESC heterogeneity is more structured than previously assumed. The advent of highly multiplexed smFISH [23, 24] offers the exciting possibility to work in a much higher state space, thereby identifying new ESC states and state interconversion models in which the end-point state distribution would be compatible with the pedigree of the cell population. This should allow to comprehensively delineate ESC heterogeneity.

## Self-Organization of Stem Cell Differentiation

Pluripotent ESCs can be differentiated to derivatives of the three embryonic germ layers upon exposure to the right signaling environment [12]. These cell fates can coexist in three-dimensional ESC aggregates called embryoid bodies [25]. However, embryoid bodies are poorly structured. Under some conditions, these embryoid bodies can remarkably self-organize into organoids, complex structures resembling actual organs. For example, optic cups [26], pituitary primordia [27], kidneys [28], cortical structures [29], or even “mini-brains” [30] have been obtained. Recent work by the Brivanlou and Siggia labs has shown that geometry is a defining factor controlling the appearance of germ layer fates [31]. The differentiation with bone morphogenetic protein 4 (BMP4) of human ESCs seeded on geometrically constrained surfaces induces the formation of trophectoderm, endoderm, mesoderm, and ectoderm fates organized in rings from the edge of the colony. At similar cell density, this spatial pattern is measured from the edge of the colony and the inner fates only appear if the colony is large enough. In a recent study, Etoc et al. [32••] combine experiments and modeling to derive a mechanistic understanding of this phenomenon of self-organization. The authors identify two mechanisms that contribute to the process: Transforming growth factor *ß* (TGF-*ß*) receptor localization and the BMP4-triggered expression of its inhibitor NOGGIN creating a reaction-diffusion mechanism. Human ESCs form an epithelium, and TGF-*ß* receptors are localized apically at the colony edge and low-cell density while they are localized laterally in the colony center and at high-cell density. This receptor relocalization renders cells in the center of colonies less sensitive to an apical BMP4 stimulation while cells at the edges can always sense BMP4. BMP4 triggers the phosphorylation and activation of mothers against decapentaplegic homolog 1 (SMAD1) and the expression of its inhibitor Noggin. Initially, cells activate uniformly SMAD1 (pSMAD1) upon BMP4 exposure, while pSMAD1 is repressed at 24 h in the central region and only activated at the colony edge. This leads the authors to suggest an inhibitory mechanism and indeed they show that knocking out NOGGIN leads to a uniform pSMAD1 profile loss of the central ectodermal region. The authors then develop a simple model to see if it can recapitulate in diverse conditions the spatial patterning of the colony in different fates.

Key ingredients of the models are the activation of SMAD1 in a radial position- and cell density-dependent way and the diffusion/degradation of BMP4-induced NOGGIN within the colony. Active pSMAD1 levels can be modeled by a Hill equation with mild cooperativity: pSMAD1 = *B*_f_^1.4^/(*B*_f_^1.4^ + *K* ^1.4^) in which BMP4 is sequestered by NOGGIN limiting its availability to the free BMP4 pool *B*_f_. The Hill constant *K* is a function of both cell density and radial position in the colony and is experimentally measured. NOGGIN production is directly proportional to pSMAD1 levels, cell density and the number of NOGGIN mRNA molecules measured by RNA-Seq. Fast diffusion and slow degradation of NOGGIN explained the experimental profiles best. The authors are thus left in their model with a single parameter to fit, corresponding to the NOGGIN levels necessary to decrease BMP4 activity by half. Considering NOGGIN levels to be zero at the border of the colony, the obtained NOGGIN concentration profile is dome-shaped, providing highest inhibition in the middle of the colony. Coupled to the relative unavailability of TGF-*ß* receptors in the center of the colony due to their lateral localization at high-cell density, this effectively shuts down the response to BMP4. From this simple model, the authors can compute the spatiotemporal profiles of pSMAD1, in excellent agreement with the experimental data.

Finally, the authors predict the concentric fate patterns at different cell densities and NOGGIN levels. Another ingredient is added to the model because mesendoderm specification requires SMAD2 signaling. pSMAD1 induces the production of the diffusible ligand NODAL, SMAD2 levels depend in turn on NODAL levels following a Hill equation without cooperativity. The fate domains depend then in a Boolean manner on SMAD1/2 levels: in the ectoderm domain, SMAD1 and SMAD2 are off; in the mesendoderm one, SMAD1 is off and SMAD2 is on while in the trophectoderm one, SMAD1 and SMAD2 are both on. Remarkably, the ordering and relative sizes of the concentric fate rings are well recapitulated by the model under wild-type conditions and NOGGIN gain- and loss-function cases. In conclusion, a model with a single adjustable parameter can account for gastrulation-like events in the dish. It highlights the essential contributions of key players.

## Stem Cell Dynamics In Vivo

While mouse and human ESCs are well-defined cell lines, the study of adult stem cells suffers from the difficulty to identify them. Indeed, the discovery of specific stem cell marker is usually transformative in our understanding of homeostasis of the respective tissues: such markers permit lineage tracing experiments and allow the isolation of stem cells for functional assays [33–35]. Notably, quantitative lineage tracing coupled to models of stem cell renewal and differentiation has been used to successfully understand skin [36, 37] and gut [38, 39] homeostasis. Moreover, rigorous comparison of quantitative data with a theoretical model can give definite answers about the potency of stem cell populations [40]. For some organs such as the mammary gland, the stem cell population is not defined unambiguously [41]. Despite this, Scheele et al. offer a model accounting for the morphogenesis of the mammary gland [42••]. The mammary gland consists of a branched network of ducts whose morphology is very heterogeneous. The authors reconstructed complete duct trees by imaging an entire mammary gland. Surprisingly, a simple model in which proliferative terminal end buds (TEBs) stochastically terminate or bifurcate with almost equal probability fits perfectly the measured characteristics of the actual ductal networks, both for the tree topology and the branch distribution. This highlights the fact that TEBs are on average functionally equivalent. Lineage tracing experiments determined that each TEB possesses around hundred mammary stem cells (MaSCs). Despite being characterized by a heterogeneous RNA expression profile, these MaSCs are equipotent as demonstrated by an analysis of subclone sizes. Such equipotency is explained by the fact that MaSCs are well mixed upon TEB bifurcation. This simple model of stochastic branching and termination is also applicable to the embryonic kidney, suggesting that an analogous mechanism of branching morphogenesis.

## Conclusions

Recent studies combining quantitative experiments with modeling allowed to characterize the heterogeneity of mouse embryonic stem cell populations [16••] and to infer state switching from end point measurements [21••]. They offered a theoretical understanding of self-organization of fate acquisition during differentiation [32••]. They also enabled the identification of strategies used by stem cells during organ growth and homeostasis in vivo [42••]. Surprisingly, in all these cases, simple predictive models accounted for the experimental observations. In fact, stochastic processes often explained the great variability originally observed in the biological system. Moreover, the simplicity of the models suggests that few master genes or cellular functions are responsible for the implementation of self-renewal or differentiation programs in a specific case. The major difficulty lies in the identification of the relevant parameters.
